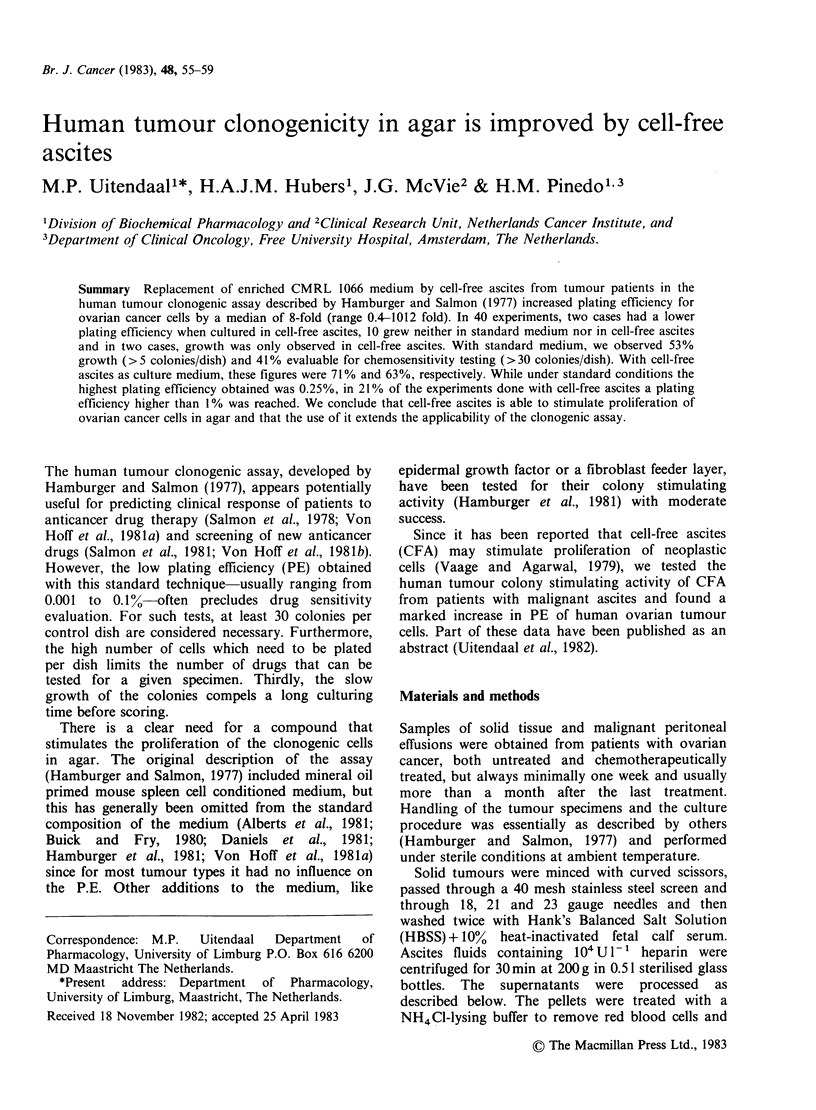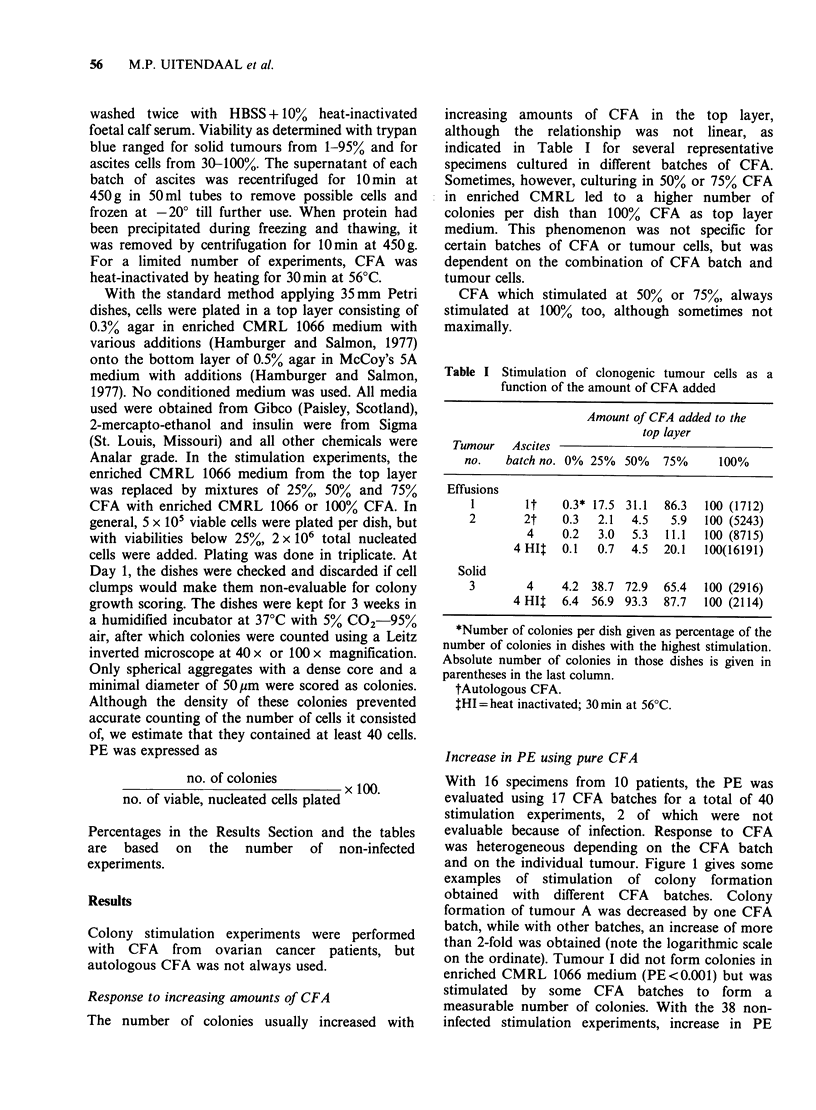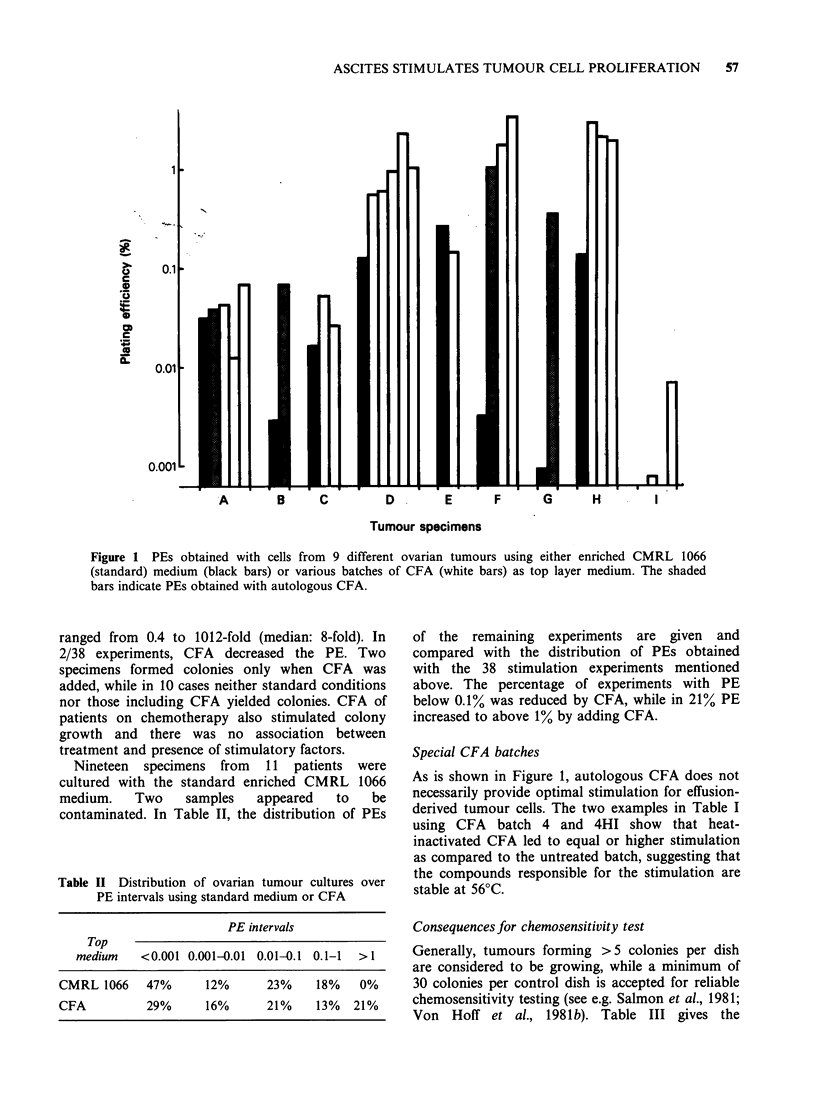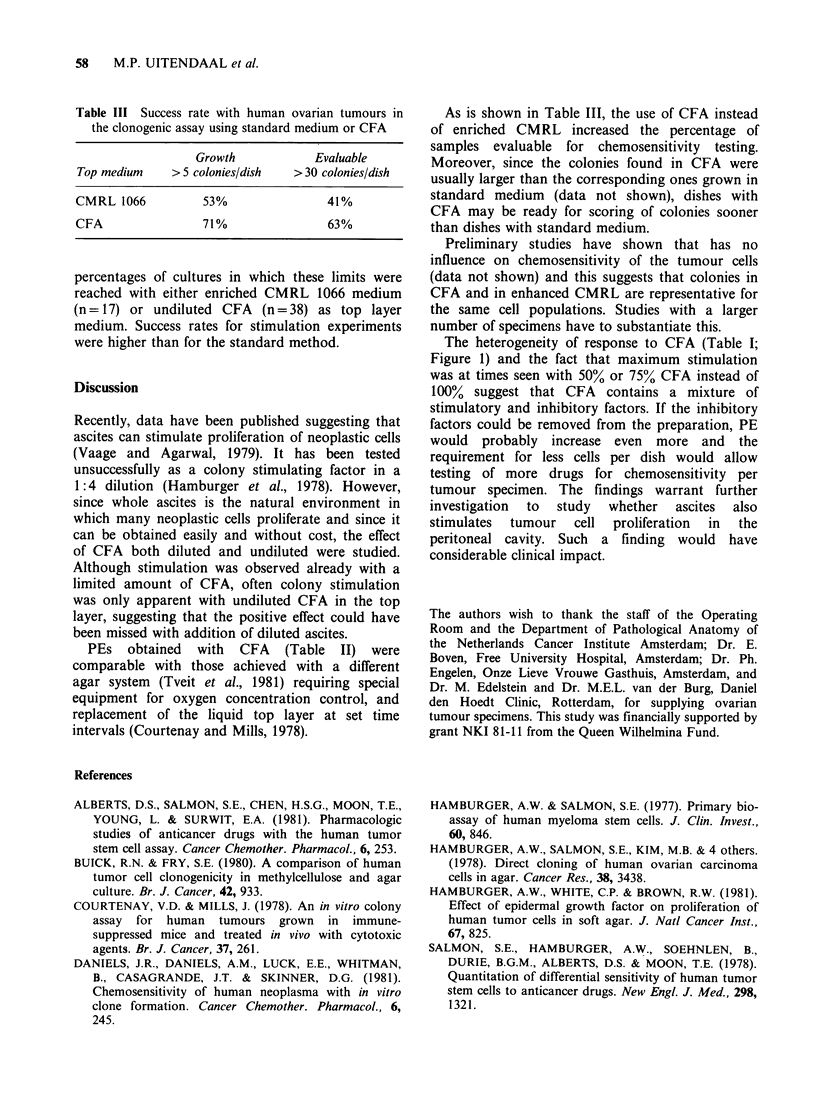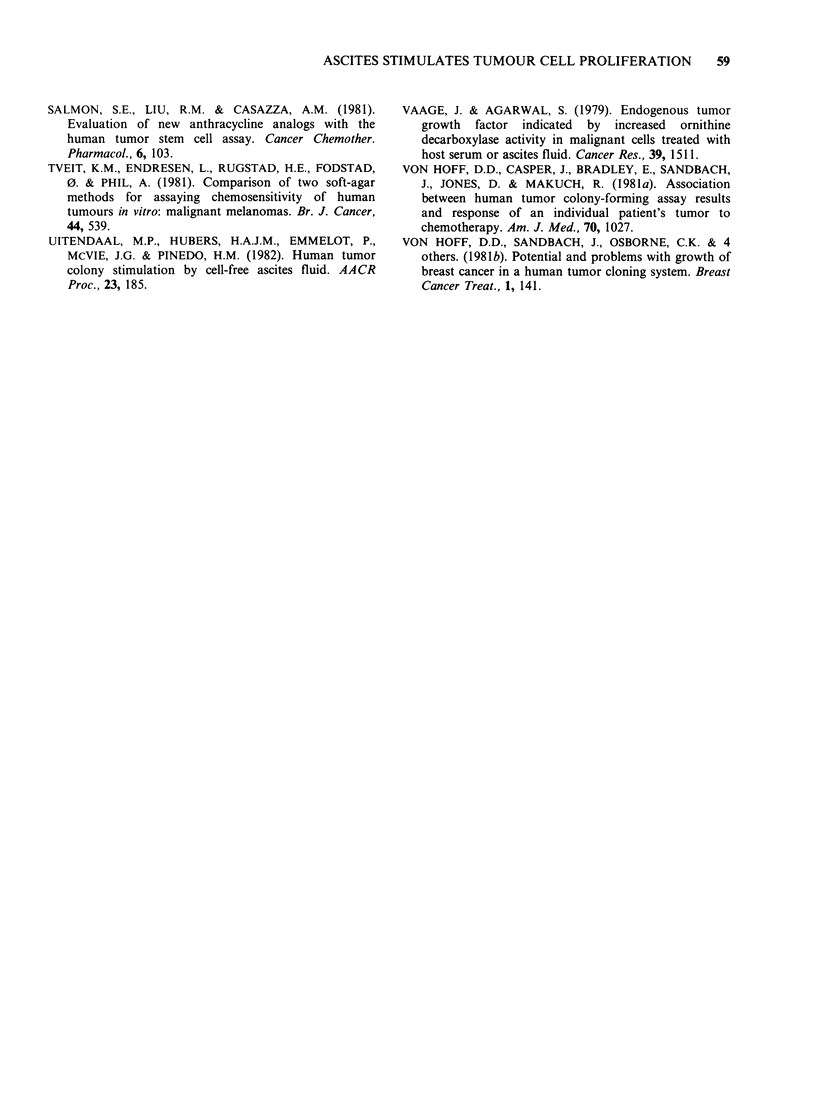# Human tumour clonogenicity in agar is improved by cell-free ascites.

**DOI:** 10.1038/bjc.1983.156

**Published:** 1983-07

**Authors:** M. P. Uitendaal, H. A. Hubers, J. G. McVie, H. M. Pinedo

## Abstract

Replacement of enriched CMRL 1066 medium by cell-free ascites from tumour patients in the human tumour clonogenic assay described by Hamburger and Salmon (1977) increased plating efficiency for ovarian cancer cells by a median of 8-fold (range 0.4-1012 fold). In 40 experiments, two cases had a lower plating efficiency when cultured in cell-free ascites, 10 grew neither in standard medium nor in cell-free ascites and in two cases, growth was only observed in cell-free ascites. With standard medium, we observed 53% growth (greater than 5 colonies/dish) and 41% evaluable for chemosensitivity testing (greater than 30 colonies/dish). With cell-free ascites as culture medium, these figures were 71% and 63%, respectively. While under standard conditions the highest plating efficiency obtained was 0.25%, in 21% of the experiments done with cell-free ascites a plating efficiency higher than 1% was reached. We conclude that cell-free ascites is able to stimulate proliferation of ovarian cancer cells in agar and that the use of it extends the applicability of the clonogenic assay.


*Br. J. Cancer* (1983), **48**, 55–59

### Human tumour clonogenicity in agar is improved by cell-free ascites

M.P. Uitendaal[1]*, H.A.J.M. Hubers[1], J.G. McVie[2] & H.M. Pinedo[1,3]

[1]*Division of Biochemical Pharmacology and* [2]*Clinical Research Unit, Netherlands Cancer Institute, and* [3]*Department of Clinical Oncology, Free University Hospital, Amsterdam, The Netherlands.*

**Summary** Replacement of enriched CMRL 1066 medium by cell-free ascites from tumour patients in the human tumour clonogenic assay described by Hamburger and Salmon (1977) increased plating efficiency for ovarian cancer cells by a median of 8-fold (range 0.4–1012 fold). In 40 experiments, two cases had a lower plating efficiency when cultured in cell-free ascites, 10 grew neither in standard medium nor in cell-free ascites and in two cases, growth was only observed in cell-free ascites. With standard medium, we observed 53% growth (>5 colonies/dish) and 41% evaluable for chemosensitivity testing (>30 colonies/dish). With cell-free ascites as culture medium, these figures were 71% and 63%, respectively. While under standard conditions the highest plating efficiency obtained was 0.25%, in 21% of the experiments done with cell-free ascites a plating efficiency higher than 1% was reached. We conclude that cell-free ascites is able to stimulate proliferation of ovarian cancer cells in agar and that the use of it extends the applicability of the clonogenic assay.

The human tumour clonogenic assay, developed by Hamburger and Salmon (1977), appears potentially useful for predicting clinical response of patients to anticancer drug therapy (Salmon *et al.*, 1978; Von Hoff *et al.*, 1981*a*) and screening of new anticancer drugs (Salmon *et al.*, 1981; Von Hoff *et al.*, 1981*b*). However, the low plating efficiency (PE) obtained with this standard technique—usually ranging from 0.001 to 0.1%—often precludes drug sensitivity evaluation. For such tests, at least 30 colonies per control dish are considered necessary. Furthermore, the high number of cells which need to be plated per dish limits the number of drugs that can be tested for a given specimen. Thirdly, the slow growth of the colonies compels a long culturing time before scoring.

There is a clear need for a compound that stimulates the proliferation of the clonogenic cells in agar. The original description of the assay (Hamburger and Salmon, 1977) included mineral oil primed mouse spleen cell conditioned medium, but this has generally been omitted from the standard composition of the medium (Alberts *et al.*, 1981; Buick and Fry, 1980; Daniels *et al.*, 1981; Hamburger *et al.*, 1981; Von Hoff *et al.*, 1981*a*) since for most tumour types it had no influence on the P.E. Other additions to the medium, like epidermal growth factor or a fibroblast feeder layer, have been tested for their colony stimulating activity (Hamburger *et al.*, 1981) with moderate success.

Since it has been reported that cell-free ascites (CFA) may stimulate proliferation of neoplastic cells (Vaage and Agarwal, 1979), we tested the human tumour colony stimulating activity of CFA from patients with malignant ascites and found a marked increase in PE of human ovarian tumour cells. Part of these data have been published as an abstract (Uitendaal *et al.*, 1982).

### Materials and methods

Samples of solid tissue and malignant peritoneal effusions were obtained from patients with ovarian cancer, both untreated and chemotherapeutically treated, but always minimally one week and usually more than a month after the last treatment. Handling of the tumour specimens and the culture procedure was essentially as described by others (Hamburger and Salmon, 1977) and performed under sterile conditions at ambient temperature.

Solid tumours were minced with curved scissors, passed through a 40 mesh stainless steel screen and through 18, 21 and 23 gauge needles and then washed twice with Hank's Balanced Salt Solution (HBSS)+10% heat-inactivated fetal calf serum. Ascites fluids containing $10^4\,U\,l^{-1}$ heparin were centrifuged for 30 min at 200 g in 0.5 l sterilised glass bottles. The supernatants were processed as described below. The pellets were treated with a $NH_4Cl$-lysing buffer to remove red blood cells and

Correspondence: M.P. Uitendaal Department of Pharmacology, University of Limburg P.O. Box 616 6200 MD Maastricht The Netherlands.

*Present address: Department of Pharmacology, University of Limburg, Maastricht, The Netherlands.

Received 18 November 1982; accepted 25 April 1983

© The Macmillan Press Ltd., 1983

washed twice with HBSS+10% heat-inactivated foetal calf serum. Viability as determined with trypan blue ranged for solid tumours from 1–95% and for ascites cells from 30–100%. The supernatant of each batch of ascites was recentrifuged for 10 min at 450 g in 50 ml tubes to remove possible cells and frozen at −20° till further use. When protein had been precipitated during freezing and thawing, it was removed by centrifugation for 10 min at 450 g. For a limited number of experiments, CFA was heat-inactivated by heating for 30 min at 56°C.

With the standard method applying 35 mm Petri dishes, cells were plated in a top layer consisting of 0.3% agar in enriched CMRL 1066 medium with various additions (Hamburger and Salmon, 1977) onto the bottom layer of 0.5% agar in McCoy's 5A medium with additions (Hamburger and Salmon, 1977). No conditioned medium was used. All media used were obtained from Gibco (Paisley, Scotland), 2-mercapto-ethanol and insulin were from Sigma (St. Louis, Missouri) and all other chemicals were Analar grade. In the stimulation experiments, the enriched CMRL 1066 medium from the top layer was replaced by mixtures of 25%, 50% and 75% CFA with enriched CMRL 1066 or 100% CFA. In general, $5\times10^5$ viable cells were plated per dish, but with viabilities below 25%, $2\times10^6$ total nucleated cells were added. Plating was done in triplicate. At Day 1, the dishes were checked and discarded if cell clumps would make them non-evaluable for colony growth scoring. The dishes were kept for 3 weeks in a humidified incubator at 37°C with 5% $CO_2$—95% air, after which colonies were counted using a Leitz inverted microscope at 40× or 100× magnification. Only spherical aggregates with a dense core and a minimal diameter of 50 μm were scored as colonies. Although the density of these colonies prevented accurate counting of the number of cells it consisted of, we estimate that they contained at least 40 cells. PE was expressed as

$$\frac{\text{no. of colonies}}{\text{no. of viable, nucleated cells plated}}\times 100.$$

Percentages in the Results Section and the tables are based on the number of non-infected experiments.

### Results

Colony stimulation experiments were performed with CFA from ovarian cancer patients, but autologous CFA was not always used.

*Response to increasing amounts of CFA*

The number of colonies usually increased with increasing amounts of CFA in the top layer, although the relationship was not linear, as indicated in Table I for several representative specimens cultured in different batches of CFA. Sometimes, however, culturing in 50% or 75% CFA in enriched CMRL led to a higher number of colonies per dish than 100% CFA as top layer medium. This phenomenon was not specific for certain batches of CFA or tumour cells, but was dependent on the combination of CFA batch and tumour cells.

CFA which stimulated at 50% or 75%, always stimulated at 100% too, although sometimes not maximally.

**Table I** Stimulation of clonogenic tumour cells as a function of the amount of CFA added

| *Tumour no.* | *Ascites batch no.* | *Amount of CFA added to the top layer* 0% | 25% | 50% | 75% | 100% |
|---|---|---|---|---|---|---|
| Effusions | | | | | | |
| 1 | 1† | 0.3* | 17.5 | 31.1 | 86.3 | 100 (1712) |
| 2 | 2† | 0.3 | 2.1 | 4.5 | 5.9 | 100 (5243) |
| | 4 | 0.2 | 3.0 | 5.3 | 11.1 | 100 (8715) |
| | 4 HI‡ | 0.1 | 0.7 | 4.5 | 20.1 | 100(16191) |
| Solid | | | | | | |
| 3 | 4 | 4.2 | 38.7 | 72.9 | 65.4 | 100 (2916) |
| | 4 HI‡ | 6.4 | 56.9 | 93.3 | 87.7 | 100 (2114) |

*Number of colonies per dish given as percentage of the number of colonies in dishes with the highest stimulation. Absolute number of colonies in those dishes is given in parentheses in the last column.
†Autologous CFA.
‡HI = heat inactivated; 30 min at 56°C.

*Increase in PE using pure CFA*

With 16 specimens from 10 patients, the PE was evaluated using 17 CFA batches for a total of 40 stimulation experiments, 2 of which were not evaluable because of infection. Response to CFA was heterogeneous depending on the CFA batch and on the individual tumour. Figure 1 gives some examples of stimulation of colony formation obtained with different CFA batches. Colony formation of tumour A was decreased by one CFA batch, while with other batches, an increase of more than 2-fold was obtained (note the logarithmic scale on the ordinate). Tumour I did not form colonies in enriched CMRL 1066 medium (PE<0.001) but was stimulated by some CFA batches to form a measurable number of colonies. With the 38 non-infected stimulation experiments, increase in PE

**Figure 1** PEs obtained with cells from 9 different ovarian tumours using either enriched CMRL 1066 (standard) medium (black bars) or various batches of CFA (white bars) as top layer medium. The shaded bars indicate PEs obtained with autologous CFA.

ranged from 0.4 to 1012-fold (median: 8-fold). In 2/38 experiments, CFA decreased the PE. Two specimens formed colonies only when CFA was added, while in 10 cases neither standard conditions nor those including CFA yielded colonies. CFA of patients on chemotherapy also stimulated colony growth and there was no association between treatment and presence of stimulatory factors.

Nineteen specimens from 11 patients were cultured with the standard enriched CMRL 1066 medium. Two samples appeared to be contaminated. In Table II, the distribution of PEs of the remaining experiments are given and compared with the distribution of PEs obtained with the 38 stimulation experiments mentioned above. The percentage of experiments with PE below 0.1% was reduced by CFA, while in 21% PE increased to above 1% by adding CFA.

**Table II** Distribution of ovarian tumour cultures over PE intervals using standard medium or CFA

| Top medium | *PE intervals* | | | | |
|---|---|---|---|---|---|
| | <0.001 | 0.001–0.01 | 0.01–0.1 | 0.1–1 | >1 |
| CMRL 1066 | 47% | 12% | 23% | 18% | 0% |
| CFA | 29% | 16% | 21% | 13% | 21% |

### *Special CFA batches*

As is shown in Figure 1, autologous CFA does not necessarily provide optimal stimulation for effusion-derived tumour cells. The two examples in Table I using CFA batch 4 and 4HI show that heat-inactivated CFA led to equal or higher stimulation as compared to the untreated batch, suggesting that the compounds responsible for the stimulation are stable at 56°C.

### *Consequences for chemosensitivity test*

Generally, tumours forming >5 colonies per dish are considered to be growing, while a minimum of 30 colonies per control dish is accepted for reliable chemosensitivity testing (see e.g. Salmon *et al.*, 1981; Von Hoff *et al.*, 1981*b*). Table III gives the

**Table III** Success rate with human ovarian tumours in the clonogenic assay using standard medium or CFA

| *Top medium* | *Growth* >5 *colonies/dish* | *Evaluable* >30 *colonies/dish* |
|---|---|---|
| CMRL 1066 | 53% | 41% |
| CFA | 71% | 63% |

percentages of cultures in which these limits were reached with either enriched CMRL 1066 medium (n = 17) or undiluted CFA (n = 38) as top layer medium. Success rates for stimulation experiments were higher than for the standard method.

### Discussion

Recently, data have been published suggesting that ascites can stimulate proliferation of neoplastic cells (Vaage and Agarwal, 1979). It has been tested unsuccessfully as a colony stimulating factor in a 1:4 dilution (Hamburger *et al.*, 1978). However, since whole ascites is the natural environment in which many neoplastic cells proliferate and since it can be obtained easily and without cost, the effect of CFA both diluted and undiluted were studied. Although stimulation was observed already with a limited amount of CFA, often colony stimulation was only apparent with undiluted CFA in the top layer, suggesting that the positive effect could have been missed with addition of diluted ascites.

PEs obtained with CFA (Table II) were comparable with those achieved with a different agar system (Tveit *et al.*, 1981) requiring special equipment for oxygen concentration control, and replacement of the liquid top layer at set time intervals (Courtenay and Mills, 1978).

As is shown in Table III, the use of CFA instead of enriched CMRL increased the percentage of samples evaluable for chemosensitivity testing. Moreover, since the colonies found in CFA were usually larger than the corresponding ones grown in standard medium (data not shown), dishes with CFA may be ready for scoring of colonies sooner than dishes with standard medium.

Preliminary studies have shown that has no influence on chemosensitivity of the tumour cells (data not shown) and this suggests that colonies in CFA and in enhanced CMRL are representative for the same cell populations. Studies with a larger number of specimens have to substantiate this.

The heterogeneity of response to CFA (Table I; Figure 1) and the fact that maximum stimulation was at times seen with 50% or 75% CFA instead of 100% suggest that CFA contains a mixture of stimulatory and inhibitory factors. If the inhibitory factors could be removed from the preparation, PE would probably increase even more and the requirement for less cells per dish would allow testing of more drugs for chemosensitivity per tumour specimen. The findings warrant further investigation to study whether ascites also stimulates tumour cell proliferation in the peritoneal cavity. Such a finding would have considerable clinical impact.

The authors wish to thank the staff of the Operating Room and the Department of Pathological Anatomy of the Netherlands Cancer Institute Amsterdam; Dr. E. Boven, Free University Hospital, Amsterdam; Dr. Ph. Engelen, Onze Lieve Vrouwe Gasthuis, Amsterdam, and Dr. M. Edelstein and Dr. M.E.L. van der Burg, Daniel den Hoedt Clinic, Rotterdam, for supplying ovarian tumour specimens. This study was financially supported by grant NKI 81-11 from the Queen Wilhelmina Fund.

### References

ALBERTS, D.S., SALMON, S.E., CHEN, H.S.G., MOON, T.E., YOUNG, L. & SURWIT, E.A. (1981). Pharmacologic studies of anticancer drugs with the human tumor stem cell assay. *Cancer Chemother. Pharmacol.*, **6**, 253.

BUICK, R.N. & FRY, S.E. (1980). A comparison of human tumor cell clonogenicity in methylcellulose and agar culture. *Br. J. Cancer*, **42**, 933.

COURTENAY, V.D. & MILLS, J. (1978). An *in vitro* colony assay for human tumours grown in immune-suppressed mice and treated *in vivo* with cytotoxic agents. *Br. J. Cancer*, **37**, 261.

DANIELS, J.R., DANIELS, A.M., LUCK, E.E., WHITMAN, B., CASAGRANDE, J.T. & SKINNER, D.G. (1981). Chemosensitivity of human neoplasma with *in vitro* clone formation. *Cancer Chemother. Pharmacol.*, **6**, 245.

HAMBURGER, A.W. & SALMON, S.E. (1977). Primary bioassay of human myeloma stem cells. *J. Clin. Invest.*, **60**, 846.

HAMBURGER, A.W., SALMON, S.E., KIM, M.B. & 4 others. (1978). Direct cloning of human ovarian carcinoma cells in agar. *Cancer Res.*, **38**, 3438.

HAMBURGER, A.W., WHITE, C.P. & BROWN, R.W. (1981). Effect of epidermal growth factor on proliferation of human tumor cells in soft agar. *J. Natl Cancer Inst.*, **67**, 825.

SALMON, S.E., HAMBURGER, A.W., SOEHNLEN, B., DURIE, B.G.M., ALBERTS, D.S. & MOON, T.E. (1978). Quantitation of differential sensitivity of human tumor stem cells to anticancer drugs. *New Engl. J. Med.*, **298**, 1321.

SALMON, S.E., LIU, R.M. & CASAZZA, A.M. (1981). Evaluation of new anthracycline analogs with the human tumor stem cell assay. *Cancer Chemother. Pharmacol.*, **6**, 103.

TVEIT, K.M., ENDRESEN, L., RUGSTAD, H.E., FODSTAD, Ø. & PHIL, A. (1981). Comparison of two soft-agar methods for assaying chemosensitivity of human tumours *in vitro*: malignant melanomas. *Br. J. Cancer*, **44**, 539.

UITENDAAL, M.P., HUBERS, H.A.J.M., EMMELOT, P., MCVIE, J.G. & PINEDO, H.M. (1982). Human tumor colony stimulation by cell-free ascites fluid. *AACR Proc.*, **23**, 185.

VAAGE, J. & AGARWAL, S. (1979). Endogenous tumor growth factor indicated by increased ornithine decarboxylase activity in malignant cells treated with host serum or ascites fluid. *Cancer Res.*, **39**, 1511.

VON HOFF, D.D., CASPER, J., BRADLEY, E., SANDBACH, J., JONES, D. & MAKUCH, R. (1981*a*). Association between human tumor colony-forming assay results and response of an individual patient's tumor to chemotherapy. *Am. J. Med.*, **70**, 1027.

VON HOFF, D.D., SANDBACH, J., OSBORNE, C.K. & 4 others. (1981*b*). Potential and problems with growth of breast cancer in a human tumor cloning system. *Breast Cancer Treat.*, **1**, 141.